# Hydrostatic reduction of iatrogenic duodenal intussusception during biliary drain exchange: A case report

**DOI:** 10.1016/j.radcr.2026.06.169

**Published:** 2026-07-22

**Authors:** Jillian E. O’Carroll, Hugo C. Temperley, Conor Keady, William Torreggiani

**Affiliations:** a School of Medicine, University College Dublin, Dublin, Ireland; b Trinity College Dublin, St James’s Cancer Institute, Dublin, Ireland; c Department of Radiology, St James’s Hospital, Dublin, Ireland; d Department of Radiology, Tallaght University Hospital, Dublin, Ireland

**Keywords:** Intussusception, Duodenal intussusception, Iatrogenic, Biliary drainage, Fluoroscopy, Hydrostatic reduction

## Abstract

Intussusception of the duodenum caused by internal/external biliary drain exchange is extremely rare but potentially life-threatening. If unrecognized, it may rapidly progress to bowel ischemia, necrosis and perforation, and thus is an important complication to recognize and treat immediately. We present the case of a 62 years old man with unresectable duodenal adenocarcinoma involving the common bile duct, managed with palliative internal/external biliary drain. During routine exchange under fluoroscopy, a duodenal intussusception was identified when contrast injection revealed a filling defect in the third part of the duodenum. The presumed mechanism was traction on the inner mucosa during retraction of the catheter. Prompt reduction of the intussusception was safely and successfully performed by rapidly injecting saline and contrast into the duodenum, avoiding complications. The patient recovered well and was discharged later that day. This case highlights a rare but important complication of biliary drain exchange and underscores the value of real-time fluoroscopic recognition and management. It is essential that clinicians performing these procedures are aware of this potential complication to ensure patient safety. To our knowledge, this is the first reported case of iatrogenic intussusception during biliary drain exchange managed successfully with hydrostatic reduction.

## Introduction

Intussusception occurs when a portion of the bowel folds into the lumen of an adjacent segment, with the prolapsed bowel termed the intussusceptum and the receiving segment of bowel described as the intussuscipiens [1,2]. This complication is rare in adults making up approximately 1% of all small bowel obstructions, with 90% caused by a pathological lead point, the most common of which is a tumor [[1], [2], [3], [4]]. It is believed that any abnormality in the bowel lumen which alters peristaltic activity can act as a lead point and cause the invagination of 1 segment of bowel into another [2]. This results in bowel obstruction and inflammation which can lead to ischemia. Moreover, iatrogenic causes are extremely infrequent with few described in the medical literature [5]. Internal/external biliary drain placement is a well-established palliative intervention to malignant biliary obstruction. Maintaining external access is advantageous, allowing quick and relatively straightforward treatment of drain complications such as drain blockage or occlusion [[6], [7], [8]]. These palliative techniques have been found to improve quality of life and prolong longevity [9]. Here, we present the case of a patient who underwent routine internal/external biliary drain exchange and subsequently developed an iatrogenic, catheter-induced duodenal intussusception successfully treated with hydrostatic reduction.

To our knowledge, this is the first reported case of duodenal intussusception occurring during internal/external biliary drain exchange and successfully managed with immediate hydrostatic reduction under fluoroscopy.

## Case report

A 62 years old man was referred to the Interventional Radiology Department for an internal/external biliary drain exchange, on a background of unresectable duodenal adenocarcinoma. The locally invasive neoplasm involved the first part of the duodenum (D1) and extended into the common bile duct and the right hepatic duct, thus requiring a palliative internal/external biliary drain.

The procedure was performed under fluoroscopy with a plan for routine exchange of an indwelling 8.5 French Cook internal/external biliary drain (Cook Medical, Bloomington, IN). A control image was obtained to assess the position of the indwelling drain. An Advantage Glidewire with a hydrophilic tip (Terumo Corp., Somerset, NJ) was placed through the indwelling drain into the duodenum and the internal/external drain was removed over the wire. A new 8.5 French drain was subsequently placed over the wire with its distal tip in the duodenum (Fig. 1). During the procedure, the distal locking pigtail loop appeared to be mispositioned in the third part of the duodenum (Fig. 2) and proximal retraction of the drain was performed. Increased resistance was felt during retraction, and retraction was halted to allow a repeat contrast injection to assess the drain position which was then appropriately positioned in the second part of the duodenum. Subsequent contrast injection demonstrated a 3 cm filling defect within the third part of the duodenum with evidence of obstruction of contrast flow beyond this area, the features of which were recognized as an intussusception (Fig. 3). The intussusception was presumed to result from traction of the catheter tip on the inner mucosa during proximal positioning. Notably, the mechanism of this case differs from typical antegrade intussusception, as the tip of the catheter pulled the distal bowel proximally.

**Fig. 1:**
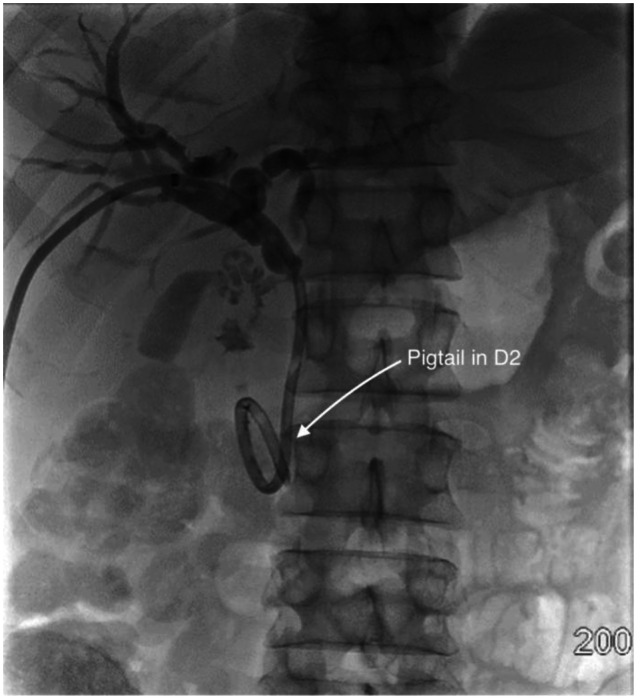
Fluoroscopic acquisition demonstrating appropriate position of the original internal/external drain prior to exchange, with the locking pigtail loop (*arrow*) sited in the second part of the duodenum (D2).

**Fig. 2:**
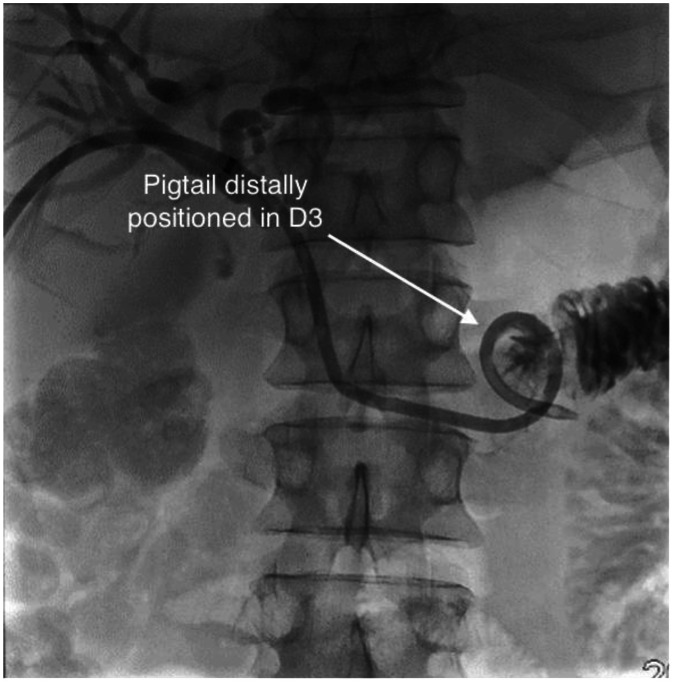
Fluoroscopic acquisition demonstrating the new internal/external drain following exchange with its locking pigtail loop (*arrow*) mispositioned distally in the third part of the duodenum (D3), prompting proximal reposition.

**Fig. 3:**
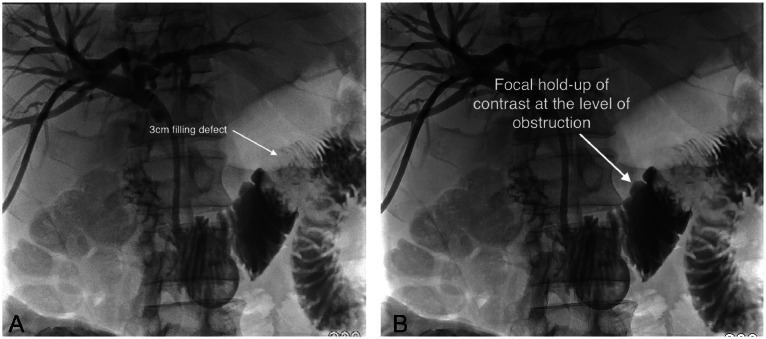
(A) Fluoroscopic acquisitions demonstrating a 3 cm intraluminal filling defect (*arrow*) in the third part of the duodenum. (B) Focal hold up of contrast at the level of obstruction (*arrow*) indicated partial luminal obstruction, consistent with an intussusception.

The intussusception was promptly treated by rapidly instilling a combination of saline and contrast into the duodenum through the internal/external drain while screening with fluoroscopy. Using a 50 mL syringe, a mixture of 50:50 saline and contrast was manually injected over 20 seconds in firm boluses. The contrast allowed real time fluoroscopic visualization of intraluminal pressure changes and reduction of the intussusception (Fig. 4). Injection was performed steadily, with the operator keeping alert to signs of perforation. No prophylactic antibiotics were used. No sedation was necessary beyond standard procedural analgesia, and bowel rest was not required. Following procedure, cross sectional imaging with computed tomography (CT) was performed to confirm that the intussusception had completely resolved. The patient remained well and was discharged later that day.

**Fig. 4:**
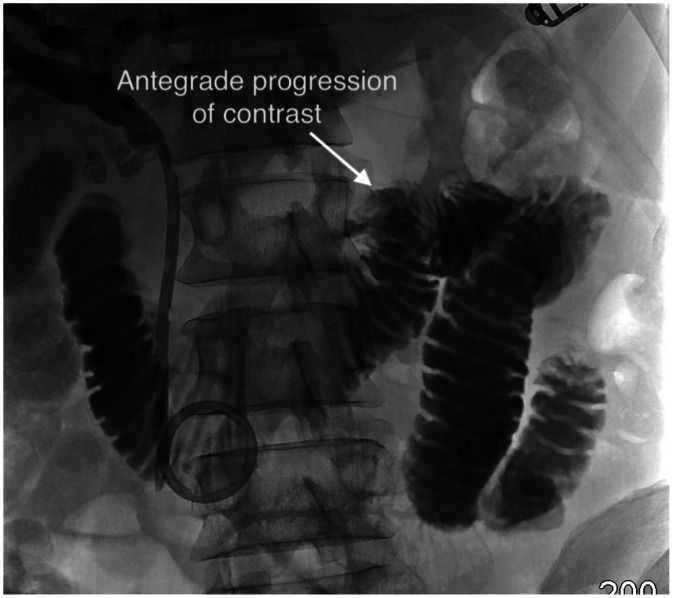
Fluoroscopic acquisition demonstrating antegrade progression of contrast (*arrow*) indicating resolution following hydrostatic reduction of the previously identified filling defect.

## Discussion

Duodenal intussusception in adults is rare, with iatrogenic causes infrequently described in the literature [[1], [2], [3], [4], [5]]. Adult intussusception is often associated with a pathological lead point, usually a malignant mass, which is in contrast to pediatric intussusception which is typically idiopathic [[1], [2], [3]].

In our case, the patient had a known duodenal carcinoma at D1 that extended into the common bile duct and right hepatic duct. Fluoroscopic imaging revealed a 3 cm discrete, round, smooth filling defect at D3, with accumulation of contrast identified proximal to the defect. While a configuration resembling the classic “coiled spring” or “bowel-within-bowel” appearance was identified on fluoroscopy, these morphologies are better visualized on CT. The discrete, fixed nature of the defect with proximal accumulation of contrast favored true intussusception over transient mucosal invagination. The intussusception is presumed to result from traction exerted by the catheter tip on the inner mucosa during proximal positioning. Notably, the mechanism of this case differs from typical antegrade intussusception, whereby normal bowel peristalsis causes invagination of the proximal bowel into a distal part. Here, proximal retraction of the catheter tip likely caused the distal bowel to be pulled proximally, resulting in an intussusception. The patient’s underlying duodenal carcinoma may have contributed to the development of the intussusception. Local tumor infiltration can alter duodenal motility, bowel wall compliance and create focal fixation points. These factors may have increased susceptibility to invagination when catheter traction was applied.

A number of differential diagnoses should be considered when an intraluminal filling defect is encountered during fluoroscopic drain exchange. These include transient mucosal folding, mucosal drag, air bubbles or intraluminal tumor extension. In this case the diagnosis of intussusception was supported by a number of features: fixed, smooth 3 cm filling defect at D3, proximal accumulation of contrast consistent with obstruction, and immediate resolution of defect following hydrostatic reduction.

Iatrogenic intussusception has been reported in a limited number of cases and is usually associated with enteral feeding devices such as percutaneous endoscopic gastrostomy, jejunostomy and long intestinal tubes [5,10,11]. In these cases the device acts as a mechanical lead point or causes an intussusception by traction during insertion or removal. A rare case of intussusception has also been described following ureteroscopic procedure, where intervention was considered the likely cause [12]. However, reported urological intussusceptions are more commonly secondary to pathological lead points such as masses or calculi rather than procedural intervention [13,14]. Both enteral-tube and urological related cases are typically diagnosed following symptom onset with cross sectional imaging, requiring endoscopic or surgical intervention. Postoperative intussusception represents another distinct subgroup, typically occurring at sites of anastomosis, where adhesions or structural changes act as predisposing factors for telescoping [15,16]. These cases and their management differ from device-related mechanisms. Reported procedure-related intussusceptions are summarized in Table 1, highlighting the difference in mechanisms, imaging modality and management approach.

**Table 1:** Procedure-related iatrogenic intussusceptions in adults: a summary of device related cases, mechanisms and management.

Study	Indication	Location	Imaging modality	Mechanism	Management	Outcome
Ishii et al. [5]	PEJ tube feeding	Small bowel	CT	Mechanical lead point	Tube removal and ileus draining	Resolution
Hu et al. [10]	Long intestinal tube removal	Small bowel	CT	Traction-induced	Manual reduction	Resolution
Zhang et al. [12]	Gastrojejunostomy tube	Small bowel/duodenum	CT	Catheter traction	Endoscopic reduction	Resolution
Present case	Biliary drain exchange	Duodenum	Fluoroscopy	Catheter traction	Hydrostatic reduction	Immediate resolution

Endoscopic reduction was not pursued in this case as the intussusception was identified and managed using real time under fluoroscopy, without the delay that endoscopy would have caused. Furthermore, the hydrostatic approach offered an immediate, minimally-invasive approach using equipment already in situ—the biliary drain, through which contrast and saline could be injected directly. Endoscopy would be a reasonable approach if fluoroscopic reduction fails or when the diagnosis is uncertain and direct visualization is needed.

While hydrostatic reduction is well established in pediatric practice, its use in adults is limited due to pathological lead points [2,17]. The anatomical distinction, lack of fixed bowel lesion and characteristic fluoroscopic imaging and prompt resolution following hydrostatic reduction collectively supports the diagnosis of an acute, catheter-induced intussusception, in the absence of a fixed pathological lead point. This suggests that hydrostatic reduction could be considered in certain, procedure related adult cases.

CT imaging is highly sensitive for diagnosing intussusception, with a classic “target” lesion appearance [18]. In this case, however, the diagnosis was made using real time fluoroscopy, allowing for direct visualization and immediate management. CT of the abdomen, with oral contrast, was performed following reduction to confirm complete resolution and to exclude complications such as ischemia or perforation. The previously noted coiled-spring/bowel-within-bowel appearance was no longer present as contrast opacified the duodenum with no evidence of intussusception (Fig. 5). There was no evidence of bowel wall thickening, edema, pneumatosis or ischemic features present.

**Fig. 5:**
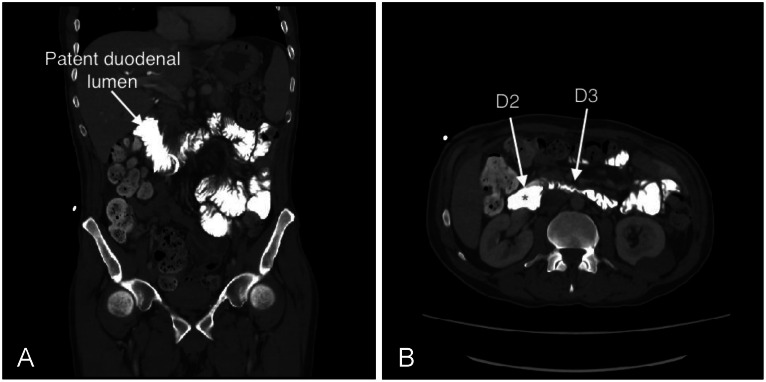
Postreduction CT of the abdomen with oral contrast, (A) coronal and (B) axial views, demonstrating resolution of the intussusception with contrast opacifying the duodenum (*arrow and asterisks*). No residual intussusception, bowel wall thickening, edema or pneumatosis identified.

This case is distinct in 3 main ways; the anatomical site (duodenum rather than jejunum or ileum), the mechanism (acute catheter traction rather than fixed lead point) and the management (immediate hydrostatic reduction). This emphasizes the importance of recognizing procedure-related intussusception, as early recognition decreases morbidity and allows for safe and effective minimally invasive management.

## Conclusion

This case demonstrates a rare iatrogenic cause of duodenal intussusception occurring during internal/external biliary drain exchange. Recognition using real-time fluoroscopy allowed for immediate diagnosis and minimally invasive management with hydrostatic reduction. Interventional radiologists should remain vigilant during such procedures, particularly in patients with altered anatomy or malignancy. Further reporting of similar cases may help refine procedural techniques and enhance patient safety.

## Patient consent

Written informed consent was obtained from the patient for publication of this case report and any accompanying images. All identifying information has been anonymized to protect patient confidentiality.
